# The role of simulator immersion on learning and transfer of decision-making skill in sport

**DOI:** 10.1007/s00426-026-02372-9

**Published:** 2026-09-03

**Authors:** Zachariah G. Hoyne, Khaya Morris-Binelli, Sean Müller, Benjamin Piggott, Paola Chivers, Evan Dekker

**Affiliations:** 1 National School of Health Sciences, The University of Notre Dame Australia, Perth, WA Australia; 2 Centre for Smart Analytics, Federation University Australia, Ballarat, VIC Australia; 3 School of Education, The University of Notre Dame Australia, Perth, WA Australia; 4 School of Medical and Health Sciences, Edith Cowan University, Joondalup, WA Australia; 5 Academic Services and Support Directorate, Federation University Australia, Ballarat, VIC Australia

## Abstract

Virtual reality has become popular in sport and other domains because it can immerse the user within a sporting context and solve logistical problems for additional off-field training. There is limited evidence, however, of whether immersion is crucial for learning and transfer. This study compared training of decision-making skill between 360-degree video virtual reality (360VR) and two-dimensional video. Twenty-eight Australian Rules Football players were randomly assigned to one of three training groups: 360VR, two-dimensional video, and control. Across four weeks, participants in the training groups were exposed to decision-making scenarios consisting of visual, contextual and auditory cues. Performance was assessed pre- and post-training with virtual reality and field-based decision-making tests. Results indicated that the two-dimensional video training group showed significantly superior decision-making in the field-based transfer test compared to 360VR and control groups post intervention. There was also indication that two-dimensional video training was superior to the control post intervention in the virtual reality test. Findings indicate that immersion created in virtual reality is not an underpinning mechanism for learning and transfer, rather the use of perceptual information is crucial. 360VR may facilitate uptake through engagement, but two-dimensional video is adequate for learning and transfer of decision-making to the field.

**Supplementary Information:**

The online version contains supplementary material available at https://doi.org/10.1007/s00426-026-02372-9.

Decision-making is a critical expert perceptual-cognitive skill in sport, which requires players to selectively attend and anticipate based upon perceptual information, then quickly and accurately make a choice response from an array of options (Müller et al. 2023; Raab et al. 2019). For example, in open play sports such as soccer, basketball, and Australian Rules Football (ARF), the decision-maker is faced with multiple teammates and opponents when required to make efficient decisions to retain or dispose of a ball. Experts have been reported to be superior at using patterns of play and direct opponent information, compared to less-skilled individuals, to make such decisions (e.g., Gorman et al. 2011; Helsen and Starkes 1999; North et al. 2009). Assessment and training of decision-making skill in open play sports is challenging given the need to organise repeated exposure for each player to multiple opponent and teammate patterns of positioning (Mangalam et al. 2023). To address this challenge, an extensive body of work has been conducted by expertise and skill learning researchers using simulators to understand the underpinning mechanism of decision-making skill, and guided by this, there is growing literature on training decision-making (Hodges et al. 2021).

Virtual reality (VR) has become popular in sport due to its immersive experience that creates a sense of presence within the simulated environment, along with ego-centric vision, and some possibility to interact with the virtual environment (Mangalam et al. 2023). Immersion is defined as the complete masking of the present world to the virtual simulation, whilst presence is defined as the subjective feeling of being transported to a simulated environment (Mütterlein 2018). In addition, there are reports from athletes, practitioners, and coaches on the value of VR simulators to augment standard practice (Dowsett et al. 2023; Mascret et al. 2022). However, it is unclear whether key features of VR such as immersion and presence are crucial to facilitate learning and transfer of decision-making to the field setting (Müller et al. 2023). Rather, it has been reported that psychological fidelity, which includes the degree to which a simulation reproduces the perceptual-cognitive information important for expert skill, is the crucial aspect for simulator content design (Harris et al. 2020). Furthermore, it is predicted as part of the modified perceptual training framework that greater correspondence between simulated domain-specific perceptual information and action coupling relative to the real-world context will facilitate transfer to the field setting (Hadlow et al. 2018). Therefore, our study presents an opportunity to determine whether a key tenant of VR in terms of immersion, as well as whether perception-action coupling, are crucial for training transfer to the real-world.

Two technological variations of VR are capable of creating immersion and presence with ego-centric vision. First, animated VR (AVR), requires marker or markerless motion capture of typical sport skills and computer programming to create avatars in a simulated environment that can be presented in a head mounted display (HMD), which the user can interact with to make decisions, as the synthetic virtual world adjusts relative to user motion (Harris et al. 2021). Second, 360-degree VR (360VR) captures video recordings of real-world scenarios using a stereoscopic camera for depth visual information (Kittel et al. 2019). Editing of captured footage is necessary to present stimulus exposures within a HMD. However, there is no possibility for interaction with the visual information because it has been previously captured and does not adjust relative to user movements within the HMD (Müller et al. 2023). Typically, both systems focus upon high physical fidelity where there is high visual resolution of the simulated world, rather than on psychological fidelity that refers to underlying sensory information that guides anticipation and decision-making skill (Harris et al. 2021; Müller et al. 2023). It is believed that high physical fidelity and immersion increases one’s sense of presence in the simulated environment, which in turn, is suggested to increase realism of the simulation relative to the natural environment and facilitate an increase in performance (Düking et al. 2018; Ochs and Sonderegger 2022; Stevens and Kincaid 2015). Despite the popularity of VR, whether these features are critical to facilitate learning of visual-perceptual motor skills, such as anticipation and decision-making, has not been investigated in sport (Müller et al. 2023).

There are fewer controlled studies that have investigated learning and transfer, rather than performance, of anticipation and/or decision-making skill using VR in sports (Faure et al. 2020). Those studies have focused primarily on the use of visual-perceptual information for anticipation and decision-making. For example, Panchuk et al. (2018) investigated whether 360VR training delivered via a HMD improved decision-making in elite youth basketballers over a three-week period. Participants were pre-and-post-tested on 360VR and small-sided games basketball specific decision-making test scenarios, with the former requiring a verbal response and latter a motor response. Participants were allocated to an intervention group who watched 15 basketball 360VR scenarios in each session, with females receiving 10 sessions and males 12 sessions over a three-week period. Both the control and intervention groups participated in routine basketball practice over the three-week period. Results revealed that male players in the intervention group showed a large, non-significant, effect size improvement in performance across 360VR (+ 4.0 points) and small-sided (+ 5.8 points) games tests, compared to the male control group (+ 0.03 and + 1.0 points for each test, respectively). For females, there were large, non-significant, effect size improvements (intervention: +7.4 points, control: +9.7 points) on the 360VR test, with only the control group showing improvement in the small-sided games test (+ 6.9 points). The authors suggested that these mixed findings could be due to inadequate statistical power and insufficient exposure to the training between male and female cohorts. Other training studies using AVR have reported improvement in heading practice to field performance in soccer (Marshall et al. 2023) and decision-making in boxing (Romeas et al. 2022). Moreover, training studies have compared the relative benefits of 360VR compared to standard two-dimensional (2D) video, with findings indicating that the former technology provided greater improvements in umpire (Kittel et al. 2020) and basketball player (Pagé et al. 2019) decision-making than the latter technology.

In the foregoing training studies, three important features involving technology and content design of VR need to be taken into consideration when evaluating their findings. First, Panchuk et al. (2018) used GoPro video cameras to capture footage and stitched them together to create a 360-degree immersive experience. This does not present stereoscopic depth visual information in the simulation. Second, comparisons made between 360VR and standard 2D video footage has not been from a comparable ego-centric perspective. Kittel et al. (2020) used broadcast footage of ARF matches that presents an elevated viewing perspective in comparison to the 360VR ego-centric perspective. Further, Pagé et al. (2019) used footage from a GoPro that presented a restricted viewing perspective for computer 2D simulation training, compared to 360-degree footage for their VR training group. Accordingly, it is not possible to directly compare the relative benefits of 360VR that are based upon immersion and presence to standard 2D video footage. Third, studies such as Pagé et al. (2019) have focused upon visual information, but have not examined learning relative to the use of contextual information. Therefore, further research is needed to carefully include these features and model multi-sensory information to obtain a more complete understanding of the benefits of VR.

While the focus has been upon visual-perceptual information, other sensory information such as contextual and auditory information is important for decision-making. In relation to contextual information, research using 2D video has reported that congruent contextual information such as opponent court positioning during tennis return of serve (Loffing and Hagemann 2014), field placings in cricket (Runswick et al. 2019), and pitch count in baseball batting (Paull and Glencross 1997), are informative for anticipation leading to decision-making. However, other studies have revealed that if performers cannot switch between incongruent contextual information and later occurring postural information, performance can be impeded (De Waelle et al. 2023; Jackson et al. 2020). In relation to auditory information, research using 2D video has reported that sound can contribute to decision-making to discriminate power of kicks in soccer and serves in volleyball (Sors et al. 2017), anticipation of stroke spin in table tennis (Park et al. 2016), as well as that congruent auditory information facilitated, but incongruent auditory information impeded, tactical decision-making in volleyball play (Klatt and Smeeton 2020). Whether the progressive inclusion of visual, contextual, and auditory information contributes to superior learning through enhanced immersion and presence created in VR, compared to standard 2D video simulation, is unknown in the literature. This is important to investigate as simulated multi-sensory information closely replicates what occurs in the natural setting. Such underpinning knowledge would help guide design and selection of simulators to accelerate performance.

The purpose of this study was to investigate whether immersion and presence maximised through muti-sensory information modelled in 360VR, compared to such multi-sensory information modelled in standard 2D video simulation, is critical to improve decision-making and transfer to the field-setting. ARF was used as the exemplar invasion sport, where participants were required to watch representative play sequences and decide which teammate they would pass the ball. In ARF, the objective is to move the ball from the team in possession’s defensive side to their attacking side of the field by passing the ball to teammates using a handpass or kick and eventually kick the ball through upright posts for a goal (Lorains et al. 2013). Based upon the evidence mentioned above, the following two hypotheses were formulated. First, it was hypothesised that 360VR and 2D video intervention groups will have superior decision-making performance across VR and field-tests after the intervention at post-test, with higher performance for the 360VR group, compared to the 2D video and control groups. Second, it was hypothesised that 360VR will have superior decision-making under multi-sensory information in the 360VR test compared to 2D video after the intervention at post-test.

## Method

### Participants

Thirty male players (*M*_age_ = 17.28, *SD* = 0.54, *M*_years played_ = 9.21, *SD* = 2.01) were recruited from a semi-professional state-level ARF team. This cohort was sampled from the highest-level of youth development competition within a state in Australia. Participants (and guardians if required) provided written informed consent, with participants providing information on whether they were colourblind, had normal or corrected-to-normal vision, if they have vertigo or other balance disorders, and if they had experienced a concussion in the previous 21 days. If participants were colourblind or had uncorrected vision they were excluded from the experiment, as this could influence use of task-related perceptual information. Likewise, if the participant had a concussion, the mandatory 21-day protocol was administered for athletes to recover, as per the ARF governing body guidelines (Australian Football League, 2024).

Ten participants (*M*_age_ = 17.05, *SD* = 0.64, *M*_years played_ = 9.30, *SD* = 2.00) who were unavailable during the pre-season period formed a naturally occurring control group. These participants were unavailable due to school scholarship commitments necessitating they prioritised school training and competition over their semi-professional club commitments during this period. The remaining twenty participants were randomly assigned using a random number table into the 360VR training group (*M*_age_ = 17.34, *SD* = 0.42, *M*_years played_ = 9.44, *SD* = 1.88) or 2D video training group (*M*_age_ = 17.46, *SD* = 0.51, *M*_years played_ = 8.90, *SD* = 2.28). All three groups shared similar levels of previous VR experience (360VR = 6 participants, 2D video = 5 participants, Control = 4 participants). Two participants withdrew from the experiment due to personal reasons and were excluded from all analyses. Of the remaining 28 participants, several missed individual testing sessions due to availability constraints which is common in youth aged athletes. In the 2D video group, one participant missed the field-based pre-test and another missed the field-based post-test. In the control group, four participants had incomplete testing data: one missed the field-based post-test, one missed the 360VR post-test, one missed both field-based tests, and one missed both 360VR tests. Participants who did not complete both pre- and post-tests were excluded from the final analyses, with the final allocation of participants as follows; VR pre-post-test (360VR, *n* = 8; 2D video, *n* = 10; control, *n* = 8), field-based transfer test (360VR, *n* = 8; 2D video, *n* = 9; control, *n* = 8), and those who completed the training (360VR, *n* = 8; 2D video, *n* = 10).

An a priori power analysis was conducted in G*Power (Version 3.1.9.6) for the comparison of decision-making performance between 360VR, 2D video, and control groups in a field-based transfer test. This analysis indicated that for a repeated measures analysis with α = 0.05, 80% power, and 95% confidence interval and a cohort of *N* = 30, a minimum of 40 trials across pre- and post-tests were required to detect a small effect (*f* = 0.16). Additionally, a priori power analysis was conducted for the comparison of decision-making performance between 360VR, 2D video, and control groups in a VR pre-post-test with a sample size of 10 participants per group (*N* = 30) and 144 trials across both testing phases, indicated a small effect size (*f* = 0.12) with α = 0.05, 80% power, and 95% confidence intervals could be detected. Finally, an a priori power analysis for the chance comparison tests in the VR pre-post-test indicated that, with a sample size of 10 participants per group (*N* = 30), a large effect size of *d* = 0.86 could be detected using a one-sample *t*-test (α = 0.05, 80% power, 95% confidence intervals, one-tailed). This allowed detection of whether mean decision-making performance were significantly above level of chance (12.5%). Ethics approval for this study was obtained from the lead authors institutional ethics committee (Reference number: 2022–140F).

### Development of decision-making pre-post tests

In ARF, the ball must be transitioned towards one’s goal in order to kick the ball through the largest of upright posts to score maximum points. The main method for this to occur is to have a teammate kick the ball to another teammate on the full without the ball landing on the ground. Once the ball is caught (i.e., marked), the player in possession of the ball cannot be impeded by opposing players within a 10 m protected area (Australian Football League, 2025). After the umpire has signalled a mark has been taken, the player in possession of the ball has approximately 6–8 s to identify a teammate to pass the ball to (either by a kick or hand) to move the ball. If by this time the player has not disposed the ball, a “play on” is called and the opposition are free to engage the player with the ball to gain possession. This mark and disposal decision was the focus of the intervention to improve performance.

#### 360-degree virtual reality test

##### Setup

**Fig. 1:**
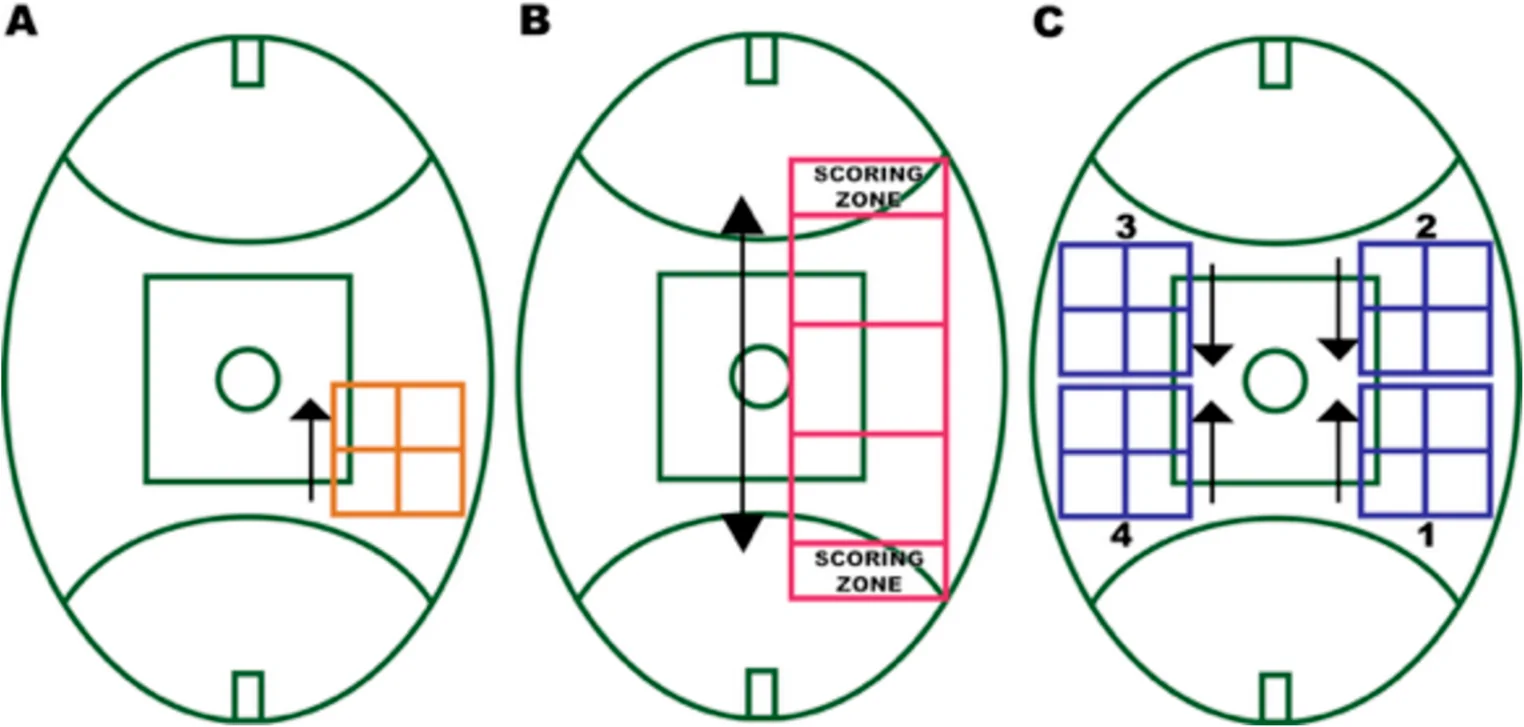
Schematic of the ARF field locations for the virtual reality pre-post-test (**A**), field-based transfer test (**B**), and 360VR and 2D video training simulus (**C**). Arrows represent direction of play. Virtual reality pre-post-test (**A**) and training stimulus (**C**) field dimensions were 30m x 30m. Field-based transfer test (**B**) dimensions were 70m *×* 40m and scoring zone 10m *×* 40m. 1, 2, 3, and 4 in C represent the different filming field locations

The 360VR test followed the procedure from Hoyne et al. (2026) and focused on the mark-disposal decision in ARF. The test consisted of eight attacking players and eight defending players running pre-determined patterns in a 30 × 30 m playing zone located on the side of an ARF regulation oval (see Fig. 1A). The playing zone was divided into four even sub-zones each containing two attacking and two defending players, with an additional defender who stood on the mark. To ensure visual differentiation in the subsequent video stimulus, attacking players wore coloured vests (orange, white, pink or yellow) whereas the defenders wore black clothing.

##### Filming

The 360VR test was filmed with a 360-degree stereoscopic video camera (Vuze 3D 360 4K VR Camera, Humaneyes Technologies Ltd., Neve Ilan Israel), sampling at 30 frames-per-second. The camera was fastened to a 1.7 m tripod approximately five meters from the intersection of the front playing zones. A resulting pool of 64 unique clips (8 running patterns × 8 unique versions), which were deemed adequate by the research team and expert coaches, were used to create the test and familiarisation trials.

##### Context and audio conditions

Contextual information was discussed and agreed upon by two expert ARF coaches (Level 3 Accredited). Contextual information was provided by the game score and time of game remaining. Two contextual conditions were created: (1) neutral context, where the participant’s team had a higher score (ahead by more than 24 points) at the beginning of the final quarter of the game, and (2) aggressive context, where the participant’s team were losing by less than one goal (6 points) with less than five minutes remaining in the final quarter of the game. The purpose of these contextual elements was to test whether participants would be sensitive to game score and time and change their decisions to a more aggressive selection of ball movement to win the game. For the audio conditions, sound information was provided which was either congruent or incongruent with the visual information. Specifically, the participant would hear a teammate instructing them to select the most correct decision or least correct decision. The audio was captured using an omnidirectional wireless microphone transmitting to a wireless receiver (RØDE GO II, RØDE Sydney, Australia). Based upon previous literature (Hoyne et al. 2026; Piggott et al. 2019), the two expert coaches discussed the scoring of each running pattern for neutral and aggressive context from best decision (8 points) to worst decision (1 point) until 100% agreement was reached (see Supplementary Material A).

##### Video sequence development

360-degree video were transferred to a laptop computer (MacBookPro 18,3, Apple Inc., California, USA) and processed in Vuze VRStudio (Version 3.4.7251, Humaneyes Technologies Ltd., Neve Ilan Israel) where they were rendered at 3840 × 2160 resolution, 30fps, 16:9 aspect ratio, H.264 codec, equirectangular over/under stereoscopic format. These videos were imported into Adobe Premiere Pro (Version 25.4.1, Adobe Inc., California, USA) to create the 360VR test. The matrix for the 360VR test consisted of 6 (running patterns) × 6 (unique versions) × 2 (sensory conditions) = 72 trials for both pre- and post-tests. In addition, six familiarisation trials were presented, consisting of each running pattern, one neutral and one aggressive context, along with four context and audio combinations. Further, six practice trials were shown including one trial each of neutral context (neutral and aggressive), neutral context with congruent and incongruent audio, and aggressive context with congruent and incongruent audio. The videos displayed in the practice and familiarisation were not used in the experiment proper.

To assess the effect of multi-sensory information on decision-making, contextual and auditory trials were blocked in a visual-contextual information condition (s1) and a visual, contextual, and auditory information condition (s2). In both conditions, contextual information in the form of game score and game time remaining were presented via a countdown timer of remaining time in the final quarter of the match and the number of points the participant’s team were ahead or behind in score for that given timepoint. The first 18 trials presented the neutral contextual information, where the participant’s team was ahead in score by more than 24 points, with the time counting down from 22 min remaining until the end of the quarter. The next 18 trials presented aggressive contextual information where the participant’s team was behind in score by less than 6 points, with less than 5 min remaining in the quarter. In s2, the addition of congruent and incongruent auditory information was presented from a virtual teammate’s verbal instruction. The congruent information instructed the participant to pass the ball to the most correct option (i.e. “Left Yellow”), whereas the incongruent instructed the worst response from available response options. The auditory information was presented in a pseudo-randomised fashion, ensuing that there was an equal spread of congruent and incongruent audio trials in both neutral and aggressive context conditions. All background audio from filming was removed in s1 condition only.

To display the trial numbers, feedback during practice trials, and contextual information, text was displayed in yellow at the front of the visual field to signify the direction of the play sequences in the visual field. Throughout the test, each trial number was displayed for three seconds, followed by the contextual information displayed for an additional three seconds (e.g., “4th QUARTER - TIME REMAINING 5:28 − 4 POINTS AHEAD”). Then, the first frame of the trial was displayed for one second to aid in orientation followed by the trial in its entirety (~ 6–8 s). An intertrial interval (ITI) of 5 s, which consisted of an audio cue after four seconds, was used to signify to participants the approach of the subsequent trial. Two separate test sequences of trials were created with the order of s1 and s2 counterbalanced across participants.

#### Field-based transfer test

##### Setup

In collaboration with one expert ARF coach (Level 3 Accredited), the researchers developed a field-based decision-making small-sided game (SSG) test focused on the mark-disposal decision. This SSG was an adaptation of the 360VR test, where a playing area of 70 m × 40 m was set up on the side of a regulation ARF oval (see Fig. 1B). An attacking team consisting of nine players who wore coloured vests were tasked with transitioning the ball from one scoring zone (extending 10 m) at the northern end of the playing field to the southern scoring end. The attacking team could transition the ball by kicking or handpassing the ball to a teammate, or by running with the ball. If the ball was successfully caught in the scoring zone, the team would then have to transition the ball to the other scoring zone straight away. To make the SSG more realistic of competition, eight defenders who wore black vests, were tasked with obstructing the offensive players from transitioning the ball. Although these defenders could tackle like in competition, if an attacking player had marked the ball, they were unable to encroach into the protected area until the umpire called “play-on”. If the ball was intercepted by a defending player, or if the ball went outside the field of play, the ball was handed back to the closest attacking player, and a defender stood on the mark of the play. Each attacking team had 2.5 min in possession of the ball, after which, the attacking and defending team swapped vests and roles. Attacking and defending groups repeated the 2.5-minute rotation eight times, as each team was given two opportunities to play in each context condition.

##### Context conditions

Like the 360VR test, two contextual conditions were created to assess whether performance changes would occur. Contextual conditions were provided to the participants in the form of game time and game score. Before the 2.5-minute rotation, all participants were made aware of the context condition on a whiteboard, which showed the neutral contextual information (“4th Quarter, 20:01 Remaining, 39 Points Ahead”) or the aggressive contextual information (“4th Quarter, 01:40 Remaining, 3 Points Behind”).

##### Scoring

The SSG was scored in a similar manner to Piggott et al. (2019), where participants were assessed on their decision-making (score). We chose to focus on decision-making score instead of skill execution or decision-making plus skill execution for the field-based transfer test, as this has been found to be the biggest contributor to prediction of successful performance in ARF athletes (Piggott et al. 2019, 2020). As in the 360VR test, the scoring of each decision was adjusted given the context condition the participants were in (see Supplementary Material B). For neutral context, scoring prioritised the most conservative option and selection of the most open teammate. In aggressive context, scoring prioritised a decision which moved the ball into the middle of the playing area whilst maintaining the direction of ball movement towards the scoring zone.

### Training intervention

#### Stimulus development

To create the training stimulus, the same running patterns from the 360VR test were re-filmed using the 360-degree video camera in three new locations as well as the original location on a standard ARF oval (see Fig. 1C), either facing north or south and on the left or right-hand side of the oval. From this, a pool of 72 training trials were created. The contextual and auditory manipulations included in the training intervention were identical to the 360VR test.

Training sequence development matched the 360VR test. However, the matrix for each training session was 2 (locations) × 4 (running patterns) × 2 (context conditions) × 2 (repeats) = 32 training trials. For each training session, the participant viewed two repeats of each running location twice per context condition (neutral and aggressive), with the order of running patterns randomised.

The sequence timing followed the 360VR test, except that following each trial, prior to the subsequent trial, the best decision was presented to participants via text for three seconds (e.g., “right white”) as feedback. Thereafter, the running pattern was replayed with the best decision written underneath the defender on the mark, so it was in the visual field of the participant but was not blocking the running patterns of the players in the video. After the participant rewatched the clip with the feedback, the trial number of the subsequent trial was presented. The training trials did not include familiarisation, nor practice, prior to any session.

The same video timeline for each training session was used for the 2D video and 360VR training groups. To ensure the visual field was in view for the 2D video, a key frame was created at the start of the trials for the researcher to zoom out to ensure the entire visual field was in view, without distorting the video.

#### Training delivery

To progressively increase the complexity of the training intervention, the presentation of contextual and auditory information changed week-to-week. During the first week, participants viewed visual-contextual information only in both training sessions. In week 2 session 1, participants viewed visual-contextual information with only congruent auditory cues, whereas in week 2 session 2, visual-contextual information with incongruent audio cues were presented. For each session of week 3 and 4, visual-contextual information with randomised congruent/incongruent audio cues were presented, ensuing an even spread of auditory information across both context conditions.

### Procedures

#### 360-degree virtual reality test

Video files were uploaded to a Meta Quest 2 HMD (Meta Platforms Inc., California, USA). Footage inside the HMD was cast and projected onto a 150” wall and recorded by a video camera with audio function (GoPro HERO9, GoPro Inc., California, USA) for later analysis. Each participant was individually assessed in the 360VR test. To help orientate participants who were new to VR, participants were given the opportunity to use the “First Steps” application that is pre-installed on the HMD to understand how to work the controllers and navigate the system. Participants were allowed five minutes in this application prior to assessment. Thereafter, participants completed the familiarisation and practice trials. During these trials, the correct response was shown, and participants were instructed to practise verbalisation of their responses.

Participants were informed that the aim of the 360VR test was to select the best response for the given trial, and that the timing of the response did not affect the scoring. Participants were instructed to respond before the next trial began, even if uncertain. On each trial, participants verbalised their decision of which teammate to pass the ball to (i.e., “left orange”, “right orange”, “left white”, “right white”, “left yellow”, “right yellow”, “left pink”, or “right pink”). Evidence indicates verbal responses are an acceptable method to assess and accelerate expert perceptual-cognitive skill, such as decision-making (Aglioti et al. 2008; Kalén et al. 2021; Müller et al. 2024a). If a participant failed to select an option, they were scored as giving the worst response (1 out of 8). To reduce cognitive fatigue, participants were provided a 5-minute break at the halfway point of the test. The time taken to complete the VR pre-post-test was approximately 25 min.

#### Field-based transfer test

To ensure each participant completed the field-based transfer test, two pre-training and two post-training tests were completed. From the pool of 30 participants, 18 were randomly selected at a time to complete the field-based test and were split into attacking and defending teams of nine participants. Two video cameras (GoPro HERO9, GoPro Inc., California, USA) positioned at each scoring end recorded the test for later coding. Prior to each test, the participants were instructed that the aim of the task was to transition the ball, like in competition, from one scoring zone (i.e., goal) to the other. They were instructed that they could kick, handpass, or run with the ball like in competition, and that the defending players could tackle and dispossess the attacking players of the ball. Following each of the 8 × 2.5-minute rotations, each team was reminded of the contextual condition (neutral or aggressive) they were in. The total time to complete one of the field-based tests was approximately 20 min.

#### Training intervention

**Fig. 2:**
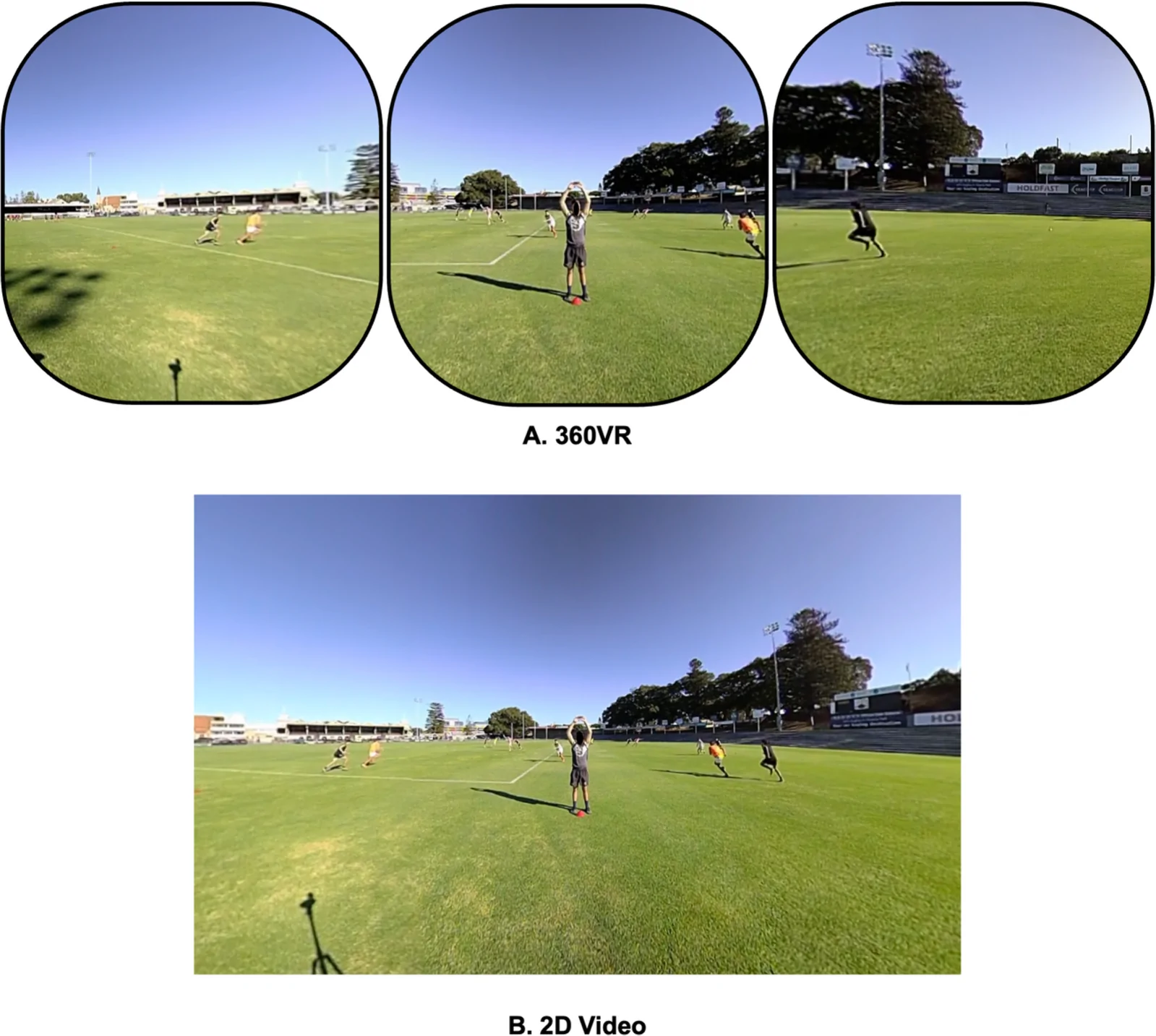
Example display of the 360VR HMD (**A**) and 2D video (**B**) conditions. The left and right still images in panel A depict the dynamic viewing perspective in the 360VR HMD whereby the user had to rotate their head in order to view the entire visual scene

Participants rotated out of on-going regular pre-season training and completed their respective training session. Following the completion of their 360VR or 2D video training session, they would return to regular training. Participants watched each session of training on their respective HMD (Fig. 2A) or projected video (Fig. 2B), where they were instructed that the purpose of training was to select the best option. The procedure matched the 360VR test, however, for the 2D video training sessions, the video files were played on a laptop computer via VLC media player (Version 3.0.18, VideoLAN Association) and projected onto a 150” wall and participants stood a mathematically calculated two meters from the projected video to ensure the same visual field was applicable across both modalities. Participants were informed to verbalise their decisions whilst a video camera (GoPro HERO9, GoPro Inc., California, USA) with audio function recorded each response for later coding. Each training session took approximately 15 min to complete.

### Dependant measures

#### 360-degree virtual reality test

Decision-making score and magnitude of change difference (post-performance – pre-performance) were the dependant variables. The independent variables were time (pre- and post-test), training group (360VR, 2D video, and Control), sensory condition (s1 and s2), context conditions within s1 and s2 (neutral and aggressive), and audio conditions within s2 (congruent and incongruent). For visualisation and interpretability, all descriptive results are the raw decision-making score (1–8) converted into a percentage of the best outcome.

#### Field-based transfer test

Decision-making score and magnitude of change difference were the dependant variables for the field-based transfer test. The independent variables were time (pre- and post-test), training group (360VR, 2D video, and Control), and context conditions (neutral and aggressive).

### Statistical analyses

Pre-test group differences were assessed using five separate Tobit regression models with training group entered as a between-group factor and participants included as a random factor: 360VR-test overall (χ^2^ (2) = 3.84, *p* =.147), 360VR-test context (χ^2^ (2) = 3.79, *p* =.150), 360VR-test audio (χ^2^ (2) = 2.03, *p* =.362), field-based overall (χ^2^ (2) = 2.09, *p* =.352) and field-based context (χ^2^ (2) = 1.05, *p* =.590). As no significant baseline differences were observed, pre-test scores were not included as covariates in the analyses. Accordingly, the focus of the subsequent analyses in testing the hypotheses was between-group differences at post-test.

Mixed-effects Tobit regression models were run in Stata (StataBE, Version 18.5, StataCorp LLC, Texas, USA) to analyse decision-making score. Tobit regression models were performed as they allow for the identification of relationships between censored dependent variables to a set of independent variables (StataCorp 2023; Williams 2016). Time (pre and post-test), training group (360VR, 2D video, Control), sensory condition (s1, context; s2, audio), as well as context (neutral, aggressive) and audio (congruent, incongruent) conditions were included as fixed effects, with individual participants included as repeated factors. Lower and upper censored limits for decision-making performance were cut-off from 1 to 8. Significant main and interaction effects were investigated using pairwise comparisons with a Holm-Bonferroni correction, which maintains control of familywise error rate while reducing the loss of power associated with standard Bonferroni adjustments (Ludbrook 1998).

In addition, linear mixed effects models (LMMs) were run in Stata to analyse the magnitude of change difference. Magnitude of change difference scores were generated by subtracting a participant’s trial level pre-test score from their post-test score. Training group (360VR, 2D video, Control) and sensory condition (s1, context; s2, audio) and the appropriate context (neutral, aggressive) or audio (congruent, incongruent) conditions were included as fixed effects, and participants included as repeated factors. Holm-Bonferroni corrections were used to assess pairwise comparisons of significant main and interaction effects. All model assumptions were checked for mixed-effects Tobit (metobit) and linear mixed-effects (mixed) analyses, and all were satisfied.

Given the unequal number of trials between pre- (*M* = 21.21, *SD* = 9.76) and post-tests (*M* = 15.96, *SD* = 5.97) in the field-based transfer test, the lower of the two trial counts was used to standardise comparisons. For each participant, only the corresponding number of trials from the larger set (pre- or post-test) were retained. For example, if a participant completed six neutral context trials at pre-test and 17 neutral context trials at post-test, only the first six post-test trials were included. This procedure ensured standardised pre-post-test comparisons across participants.

The intra-rater and inter-rater reliability of the scoring of the field-based test was assessed using linear weighted Cohen’s Kappa (LWK). As per McHugh (2012), the following benchmarks of agreement between scorers were used for LWK coefficient values: ≤ 0.20 (none), 0.21–0.39 (minimal), 0.40–0.59 (weak), 0.60–0.79 (moderate), 0.80–0.90 (strong), and > 0.90 (almost perfect). The results of these tests demonstrated strong intra-rater (LWK = 0.86, *SE* = 0.03, *p* <.001) and inter-rater (LWK = 0.84, *SE* = 0.03, *p* <.001) reliability.

For the 360VR test, decision-making performance were also compared against chance (12.5%) using one-sample *t*-tests or Wilcoxon one-sample signed-rank tests, depending on violation of normality assumptions. These comparisons assessed whether decision-making performance exceeded the level expected by chance.

## Results

### 360-degree virtual reality test

#### Overall

**Fig. 3:**
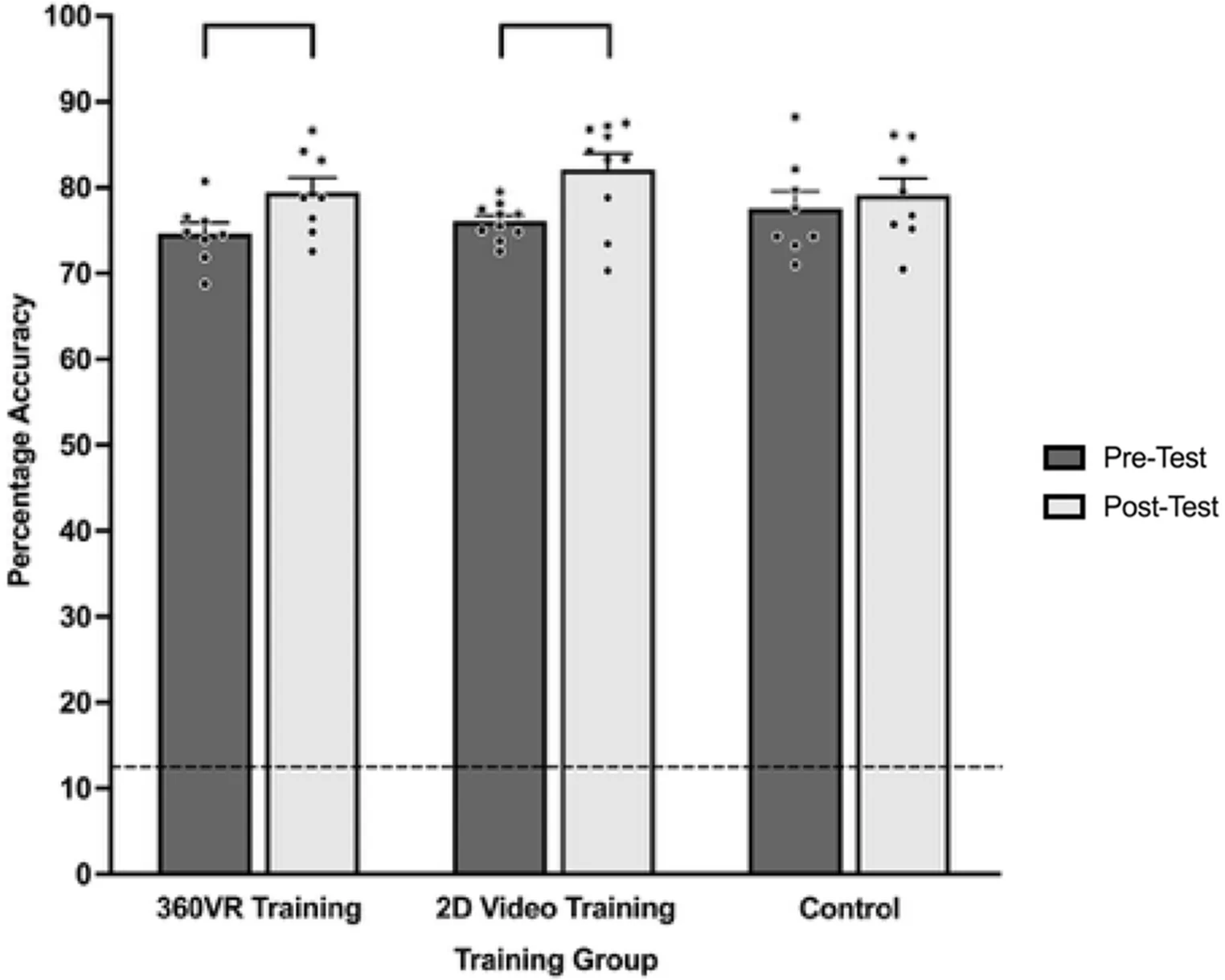
Mean decision-making accuracy in VR by training group relative to testing phase. Bracketed groupings indicate significant differences in pre-test to post-test for 360VR and 2D video training groups (*p *< .05). Dashed line represents chance-level performance (12.5%). Error bars show standard error of the mean, and individual participant responses are shown by dots

Collapsing context and audio conditions, metobit detected a significant time × training group interaction on decision-making performance, χ^2^ (2) = 6.28, *p* =.043. Post-hoc tests indicated that both the 360VR (*p* =.001) and 2D video (*p* <.001) training groups significantly improved from pre- to post-test, whilst the control group showed no significant improvement (*p* =.367), see Fig. 3. However, at post-test, no significant difference was detected between 360VR and control (*p* =.736), or between 2D video and control (*p* =.079). Performance was also not significantly different between 360VR and 2D video at post-test (*p* =.161).

Regarding magnitude of change difference, LMM did not detect a significant training group main effect with context and audio collapsed, χ^2^ (2) = 3.88, *p* =.143.

For the chance comparisons, all groups performed significantly above the level of chance (12.5%) at post-test, with large effect sizes: 360VR (*r* =.89), 2D video (*r* =.89), and control (*d* = 11.90), all *p*s < .001. At pre-test, all groups were significantly above the guessing level, *p*s < .001.

#### Context

Metobit detected a significant time × training × context (s1) interaction on decision-making score, χ^2^ (2) = 8.33, *p* =.016. For neutral context, follow-up post-hoc tests indicated that neither the 360VR (*p* =.456), 2D video (*p* =.176), or control (*p* =.701) group significantly improved from pre- to post-test. At post-test, there were no significant differences detected between 360VR (*M* = 85.94%, *SE* = 1.66%) and control (*M* = 85.07%, *SE* = 1.90%), *p* =.828, nor between 2D video (*M* = 87.01%, *SE* = 1.45%) and control, *p* =.694. There were also no significant differences detected between 360VR and 2D video (*p* =.389). For aggressive context, follow-up post-hoc tests detected no significant differences between 360VR (*M* = 73.70%, *SE* = 2.07%) and control (*M* = 73.00%, *SE* = 1.89%), *p* =.650, or between 2D video (*M* = 76.39%, *SE* = 1.94%) and control, *p* =.137. Further, no significant differences were detected between 360VR and 2D video (*p* =.312).

**Fig. 4:**
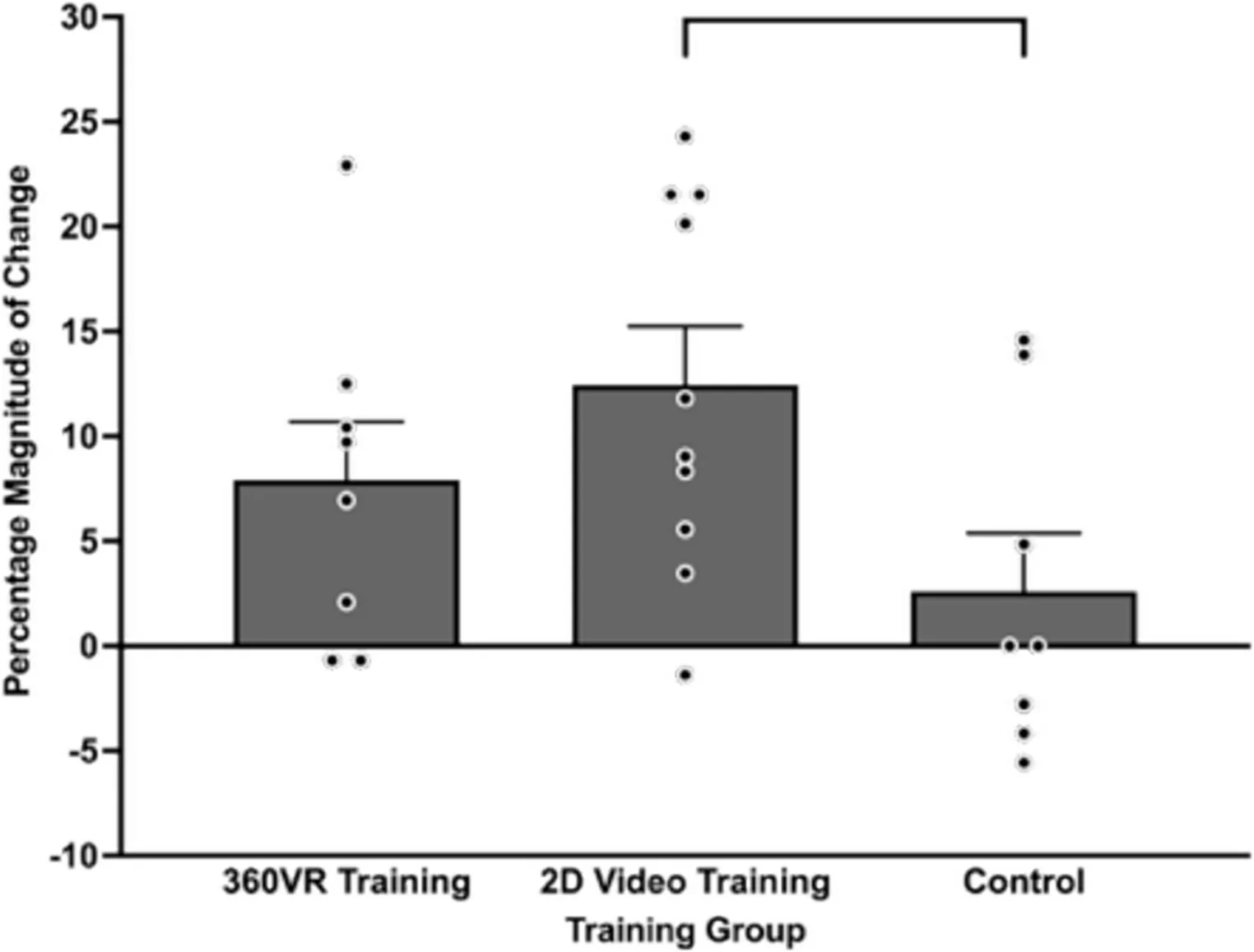
Decision-making magnitude of change difference in VR sensory 1 aggressive context by training group. Bracketed groupings indicate significant magnitude of change difference between 2D video and Control groups (*p *< .05). Error bars indicate standard error of the mean and individual participant responses are indicated by dots

LMM detected a significant training group × context (s1) interaction on magnitude of change difference, χ^2^ (2) = 8.12, *p* =.017. However, for neutral context, follow-up post-hoc comparisons did not detect a significant magnitude of change difference between 360VR (*M* = 0.95%, *SE* = 2.10%) and control (*M* = −0.26%, *SE* = 1.73%), *p* =.708, nor was a significant difference between 2D video (*M* = −2.08%, *SE* = 2.32%) and control (*p* =.554) or 360VR (*p* =.324) detected. For aggressive context, no significant difference for magnitude of change was detected between 360VR (*M* = 7.90%, *SE* = 2.36%) and control (*M* = 2.60%, *SE* = 1.92%), *p* =.103, or 2D video (*M* = 12.43%, *SE* = 2.29%), *p* =.141. There was a significant magnitude of change difference between 2D video and control, *p* =.001 (see Fig. 4).

All groups scored significantly above chance at post-test across both context conditions (neutral, aggressive), with one-sample *t*-tests (*p*s < .001) and Wilcoxon signed-rank test (*p*s = .012) demonstrating large effect sizes: 360VR (neutral, *r* =.89; aggressive, *d* = 9.10), 2D video (neutral, *d* = 13.34; aggressive, *d* = 8.25), and control (neutral, *r* =.89; aggressive, *d* = 6.22). At pre-test, all groups were significantly above the guessing level, *p*s < .05.

#### Audio

Metobit did not detect a significant time × training × audio (s2) interaction on decision-making score, χ^2^ (2) = 2.98, *p* =.226. When audio (congruent, incongruent) was collapsed, metobit did indicate a significant time × training interaction, χ^2^ (2) = 6.72, *p* =.035. Follow-up post-hoc comparisons indicated that the 2D video group significantly improved from pre- (*M* = 75.59%, *SE* = 1.42%) to post-test (*M* = 82.47%, *SE* = 1.37%, *p* <.001), whilst no significant improvement was detected for the 360VR (pre-test, *M* = 73.91%, *SE* = 1.61%; post-test, *M* = 79.04%, *SE* = 1.67%) and control group (pre-test, *M* = 77.26%, *SE* = 1.60%; post-test, *M* = 79.17%, *SE* = 1.48%), *p*s > .05. At post-test, decision-making score was not found to be significantly different between 360VR and control (*p* =.985). Also, no significant difference was found between 2D video and control (*p* =.096), and 2D video and 360VR (*p* =.096).

Regarding magnitude of change, LMM did not report a significant training × audio interaction, χ^2^ (2) = 3.84, *p* =.147.

All groups performed significantly above chance at post-test across both audio conditions (*p*s < .001) and demonstrated large effect sizes: 360VR (congruent, *r* =.89; incongruent, *r* =.89), 2D video (congruent, *d* = 11.30; incongruent, *d* = 6.31), and control (congruent, *r* =.89; incongruent, *r* =.89). At pre-test, all groups were significantly above the guessing level, *p*s < .001.

### Field-based transfer test

#### Overall

**Fig. 5:**
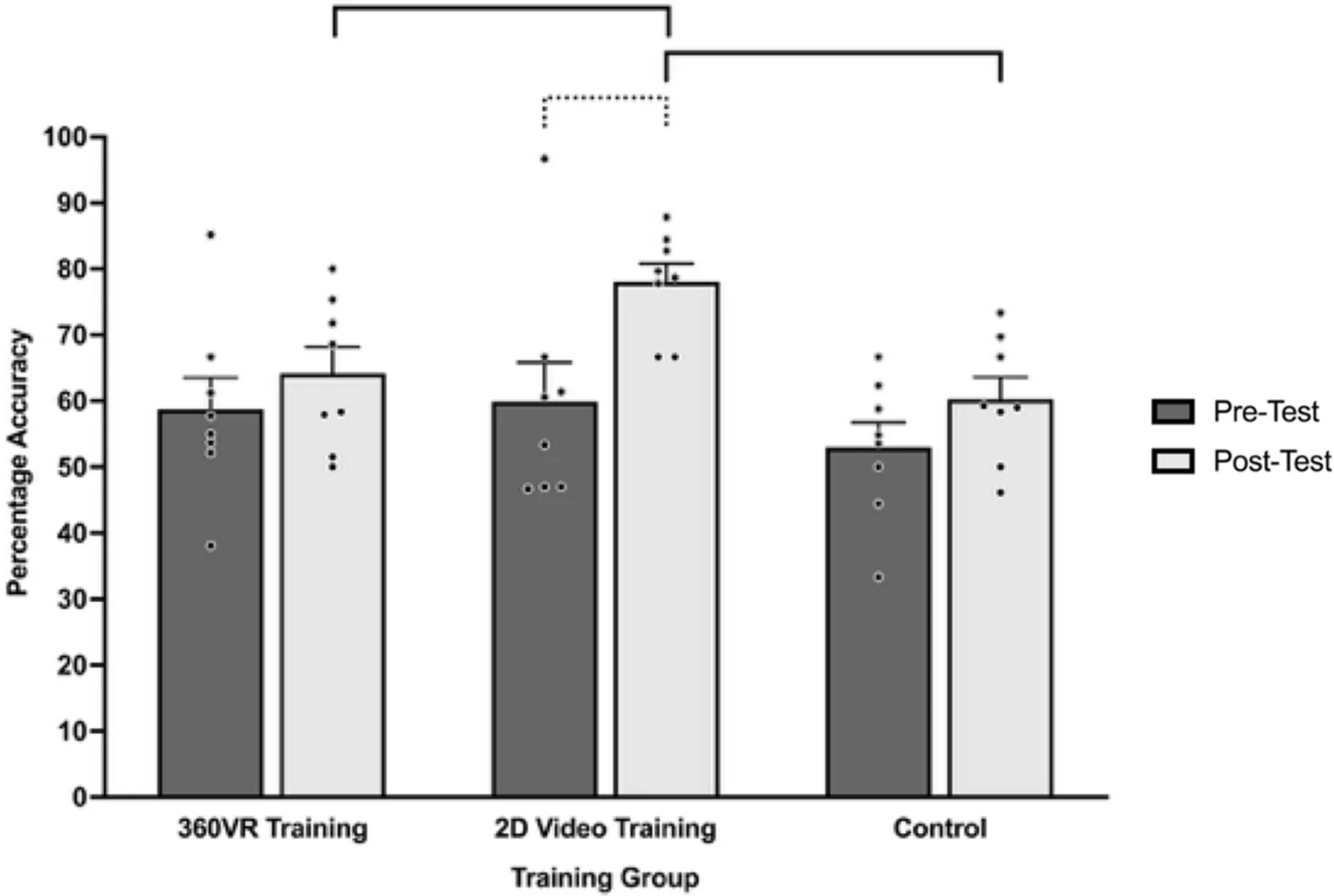
Mean decision-making accuracy in the field-based transfer test for contextual information collapsed by training group. Solid bracketed groupings indicate significant differences between 2D video and both 360VR and control at post-test. Dotted bracketed grouping highlights significant difference between pre-test and post-test for 2D video. Error bars indicate standard error of the mean and individual participant responses are indicated by dots

Metobit did not detect a significant time × training × context interaction on decision-making score, χ^2^ (2) = 4.93, *p* =.085. However, with context collapsed there was a significant time × training interaction, χ^2^ (2) = 8.53, *p* =.014. Follow-up post-hoc comparisons indicated the 2D video significantly improved from pre- to post-test (*p* <.001), whilst no significant improvement was found for 360VR (*p* =.406) and control (*p* =.251), see Fig. 5. At post-test, no significant differences were detected in decision-making score between 360VR (*M* = 63.17%, *SE* = 3.10%) and control (*M* = 58.92%, *SE* = 4.49%), *p* =.441. However, there were significant differences in decision-making score between 2D video (*M* = 78.49%, *SE* = 2.81%) and both control, *p* =.001, and 360VR (*p* =.003), see Fig. 5.

LMM did not report a significant training × context interaction on magnitude of change, χ^2^ (2) = 2.51, *p* =.286.

#### Neutral Context

**Fig. 6:**
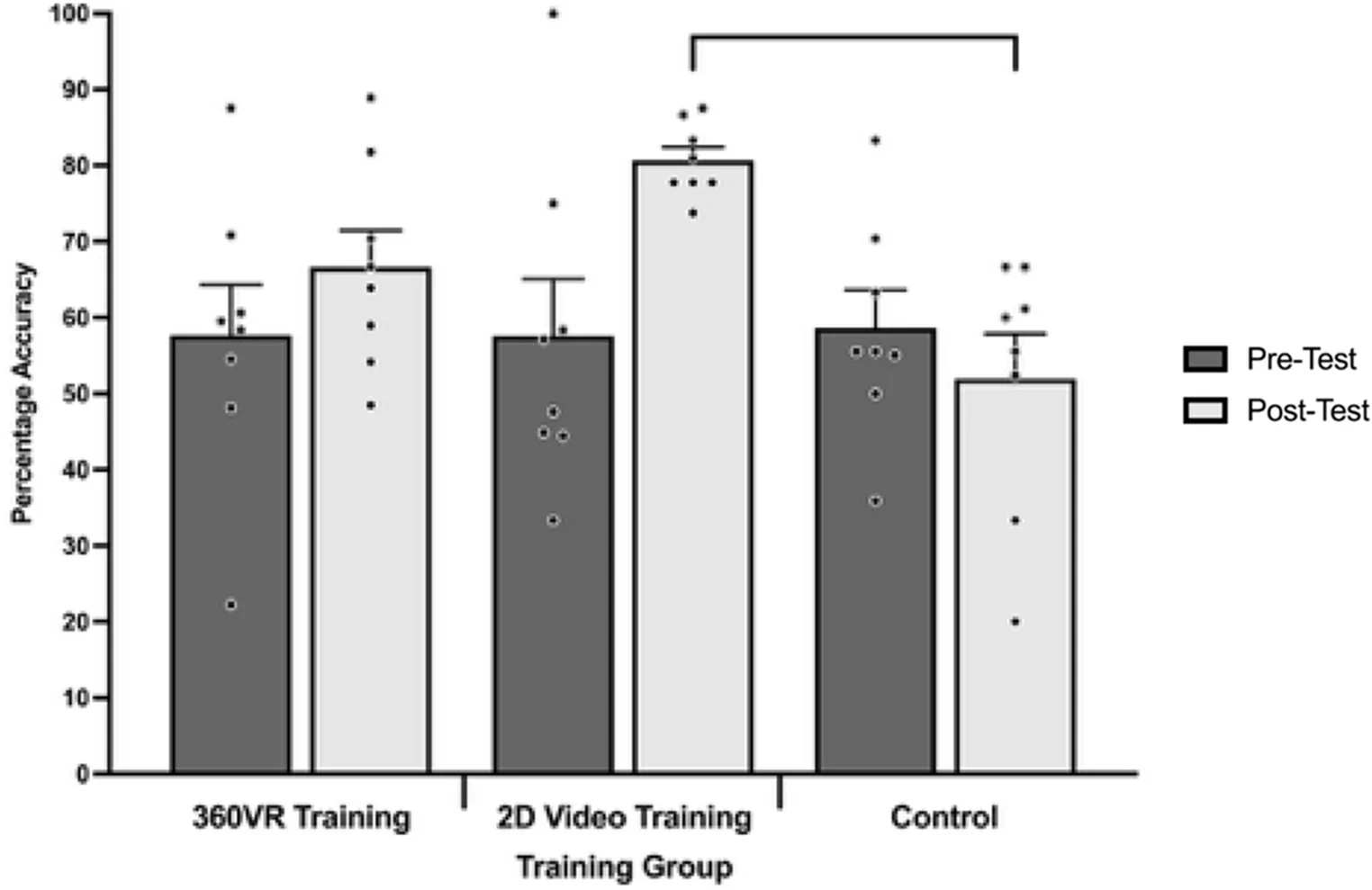
Mean decision-making accuracy in the field-based transfer test by training group in neutral context condition. Bracketed groupings indicate significant difference between 2D video and control at post-test. Error bars indicate standard error of the mean and individual participant responses are indicated by dots

Metobit model detected a significant time × training interaction on decision-making score, χ^2^ (2) = 8.33, *p* =.016. Follow-up post-hoc comparisons indicated 2D video significantly improved from pre- to post-test (*p* <.001), whereas the 360VR (*p* =.351) and control (*p* =.572) did not report a significant improvement. At post-test, no significant differences were detected in decision-making score for 360VR (*M* = 65.32%, *SE* = 5.15%) compared to control (*M* = 52.73%, *SE* = 5.91%), *p* =.061, or to 2D video (*M* = 79.45%, *SE* = 3.71%), *p* =.094. However, decision-making score was significantly higher in 2D video compared to control (*p* <.001), see Fig. 6.

LMM did not detect a significant training main effect on magnitude of change in the neutral context trials, χ^2^ (2) = 4.45, *p* =.108.

#### Aggressive Context

In aggressive context, metobit detected no significant time × training interaction on decision-making score, χ^2^ (2) = 4.07, *p* =.131. Further, LMM detected no significant training main effect on magnitude of change, χ^2^ (2) = 1.18, *p* =.556.

## Discussion

The purpose of this investigation was to determine the relative benefits of immersion and psychological fidelity, through simulation of multi-sensory information, to the enhancement of decision-making, and transfer to the field. The findings from this study support the use of 2D video simulation as a suitable option to enhance decision-making skill, compared to modern 360VR technology. In addition, the findings highlight the importance of modelling multi-sensory information in simulators to facilitate learning and transfer of decision-making skill to the field setting. In relation to hypothesis one, it was predicted that 360VR and 2D video training groups would attain superior decision-making across 360VR and field tests after the intervention at post-test, with higher performance for 360VR training, compared to the control group. The hypothesis was partially supported, as the 360VR test did not reveal any significantly superior performance by the 360VR group compared to the 2D video and control groups at the post-test (see Figs. 3, 4 and 5). However, contrary to our hypothesis, in the field-test, the 2D video training group attained decision-making performance significantly greater than both the 360VR and control groups, indicating superior transfer from laboratory training to the field (see Figs. 5 and 6). This suggests that the proposed benefit of 360VR, namely greater immersion and presence compared to traditional video simulation, may not be a crucial contributing factor to learning and transfer as previously thought.

The reason critical 360VR features of immersion and presence that masked participants from the current environment (room), and transported them to a simulated football ground, respectively, were not superior for learning and transfer, is likely because psychological fidelity of the learning process was a more crucial factor (Ochs and Sonderegger 2022). The 2D simulation in this study contained the complete patterns of structured play information including relational properties between attackers and defenders for performers to be able to make effective decisions. This is in line with a broad body of previous video-based performance (e.g., Gorman et al. 2011; Helsen and Starkes 1999) and training (e.g., Lorains et al. 2013) studies on decision-making in sport. In comparison, previous studies have presented restricted viewing angle (Pagé et al. 2019) or an elevated grandstand viewing perspective (Kittel et al. 2019) for 2D simulation training that may have limited the capability to improve use of relational information and transfer learning to ego-centric vision test contexts. A lack of available contextual information within the training 2D stimuli (e.g., Pagé et al. 2019) may have also limited efficient use of relational information for transfer to field tests. Further, the training in 360VR whereby participants’ observed patterns of play similar to being present on the football field, with the provision of depth visual information, did not contribute to accelerated performance beyond that of a two-dimensional video projected display. This lack of a need for stereoscopic depth information is interesting given that the displayed pattern of play occurred over a playing area of up to 35 m in depth. It is possible, however, that depth information was extracted from 2D sources (Miles et al. 2012), which may explain this finding.

In relation to hypothesis two, it was predicted that the 360VR training group would attain superior decision-making skill after the intervention at post-test compared to 2D video across combined simulation of multi-sensory information. Based upon the VR overall test that included visual + contextual + auditory information collapsed together, superior performance was not found for the 360VR training group compared to the 2D video training group, so this hypothesis was not supported. In contrast, with regards to isolated context conditions, there was superior performance after the intervention for the 2D training group compared to control group in the aggressive context condition, suggesting the 2D video training led to greater improvement in decision-making under more isolated sensory information, albeit with no difference to the 360VR group. It is not possible to determine the relative use of all three sensory information sources in the field test, because only visual + context was manipulated due to the challenges of including auditory conditions in the field. Nonetheless, in comparison to the 360VR test, combined, rather than isolated, sensory conditions, including both neutral and aggressive contextual information, is where the 2D video training group performance superseded both other groups in the field test post-intervention.

A possible speculative explanation for these findings is that it may be that there was sensory overload or conflict that occurred in the HMD under multi-sensory conditions. For example, in other domains, studies have reported that multi-sensory congruent, rather than incongruent, information contributed to increased presence ratings, with no decrement to performance on a naval navigation task (Glenn and Coxon 2024), whilst other studies reported no significant differences in presence or performance across congruent isolated to multi-sensory conditions in a locomotion navigation task (Lee et al. 2024). Accordingly, congruency of multi-sensory information may increase perceptions of presence to a level that it detracts from learning. Therefore, modelling multi-sensory information and its overload of feelings of presence should be taken into consideration for progressively challenging training design in VR.

The transfer of superior learning from 2D video-based simulation training to the field-based small-sided game is important to consider. This is a consistent finding that has been previously reported in the literature, where 2D video-based training conducted in laboratory settings (e.g., conference or club room) has improved field-based tennis return of serve (Smeeton et al. 2005), penalty corner saves in field hockey (Morris-Binelli et al. 2021), and open field play decision-making in soccer (Gabbett et al. 2008), to name a few. The 2D video-based training in our study, and others reported here, involved a perceptual response that was uncoupled from action. This did not limit, but rather, facilitated benefits to decision-making in the field that required a coupled perception-action response. This finding is inconsistent with the prediction of the modified perceptual training framework that states perception-action coupling is necessary for transfer (Hadlow et al. 2018). Therefore, perception-only training, which challenges decision-making skill through psychological fidelity of the 2D simulation appears to map onto action responses in the field to facilitate transfer of learning (Müller et al. 2024a).

There are potential limitations of our study that need to be taken into consideration. First, the study was focused upon one aspect of open field play sports that required the performer to make decisions from a stationary perspective of the game. Second, there was restriction on accessibility to athlete time and the need to use a naturally existing control group, which limits the length and frequency of training, as well as equal randomisation of participants to groups. It should be noted, however, due to the demanding competition, training, and work/study schedule of athletes, one-month access to implement perceptual training was reasonable and in line with previous literature (e.g., Morris-Binelli et al. 2021). To this, there is the need for balance between internal and external validity such that high adherence to both is unrealistic in an applied setting like the one in which this study was conducted (see; Müller et al. 2015). Accordingly, some flexibility and an appropriately designed scientific method is needed, such as randomisation where possible and use of naturally occurring and comparable skill level participants, to ensure studies like ours are conducted so that findings can be used to enhance learning of relevant sport populations (Müller et al. 2015). Third, participant attrition resulted in minor deviations from the planned sample size. The expected total sample size was *N*= 30, but with a drop-out of two participants attrition equated to 7%. Accordingly, in each of the three groups there were under ten participants who completed the study. Given this small sample size, caution needs to be exercised in terms of being definitive about our conclusions. Follow-up studies are needed to replicate our findings, which will then provide greater confidence to confirm or amend our findings and application of them to player training in the real-world. Such studies could use our data to calculate sample size more accurately and accommodate attrition, which will help with planning and implementation. Nonetheless, while attrition may have reduced statistical power to detect small effects, the detection of meaningful between-group differences indicates that the study retained sufficient sensitivity for the primary outcomes of interest. This means that the psychological fidelity designed into our tests was suitable to capture perceptual-cognitive-motor learning and transfer. Finally, manipulation checks of presence were not employed in this study, but it has been well established that factors including spatial presence, engagement, and ecological validity, are higher in 360VR compared to 2D video simulation (see Hoyne et al. 2026). Immersion is a technology feature of virtual reality, so it is not a measure, but is created through use of a 360VR head mounted display (Hoyne et al. 2026).

## Conclusions, future research, and practical implications

The findings from this four-week training intervention indicate that, when aiming to enhance decision-making skill, the informational context or psychological fidelity of the simulation is more important than its immersive properties. Given there was mixed benefits to 360VR training compared to 2D video simulation in improving decision-making across virtual and field-based assessments, the added complexity and cost of immersive VR should prompt practitioners to carefully evaluate its implementation value to accelerate performance. This is particularly important as high physical fidelity VR is being investigated with the view of implementation for training personnel across several domains such as in military (Harris et al. 2023), law enforcement (Guo et al. 2025), aviation (Vallès-Català et al. 2026), and air force (Kirollos et al. 2025). VR may offer unique benefits in engagement of the user (Hoyne et al. 2026), but these alone may not guarantee superior learning and transfer outcomes. Accordingly, practitioners in domains such as law enforcement should carefully consider the psychological fidelity for simulator training. For example, police shoot-no-shoot decision-making simulator design should incorporate abstraction techniques to target relational movement pattern information to challenge the fast visuomotor processes of officers’ decision-making during training (Müller 2024). This can be delivered using either 2D video or 360VR technology that is portable and lower in cost than synthetic VR simulators. Future research should compare the relative benefits of 360VR, AVR, and 2D video simulation training to other dynamic aspects of open field play sports. For example, it could be that the immersive properties of 360VR provide greater benefits to learning and transfer of decision-making in tightly contested aspects of play, where there is greater inter-player interaction required, which require a greater level of 360-degree information use than in our task. Further, the criteria for evaluating Cohen’s Kappa should be considered in future statistical analyses (McHugh 2012). In terms of practical application, as the accessibility of 360VR and AVR and other extended reality technologies continues to grow, sports science practitioners could use a combination of 360VR and 2D video to target engagement and psychological fidelity to boost motivation for learning (Wulf and Lewthwaite 2016). Collectively, simulators present a suitable complement for standard practice, where they can overcome logistical challenges of coordinating groups of players for those individuals who require additional decision-making training, which appears to address the need mentioned by players and coaches alike (Dowsett et al. 2023; Mascret et al. 2022).

## Supplementary Information

Below is the link to the electronic supplementary material.

Supplementary Material 1 (DOCX 37.3 KB)

## Data Availability

The data that support the findings of this study are available upon reasonable request from the corresponding author. The data are not publicly available as it contains information that could compromise the privacy of research participants.
