# Supplementary material for: The role of simulator immersion on learning and transfer of decision-making skill in sport
Source: Psychol Res. 2026 Sep 3;90(5):159. doi: 10.1007/s00426-026-02372-9 (PMC13541863; doi:10.1007/s00426-026-02372-9)
Supplement: Supplementary file 1 [file 426_2026_2372_MOESM1_ESM.docx]

# Supplementary Material

**Associated Manuscript**: The Role of Simulator Immersion on Learning and Transfer of Decision-Making Skill in Sport.

**Authors**: Hoyne, Z.G., Morris-Binelli, K., Müller, S., Piggott, B., Chivers, P., and Dekker, E.

**Journal**: Psychological Research

## A. Decision-Making Scoring Criteria for Neutral and Aggressive Contextual Information in the Virtual Reality Pre-Post-Test

| **Running Pattern** | **Context Condition** | **8** | **7** | **6** | **5** | **4** | **3** | **2** | **1** |
| --- | --- | --- | --- | --- | --- | --- | --- | --- | --- |
| 1 | Neutral | LW | RY | LO | RP | LP | LY | RW | RO |
|  | Aggressive | LO | LY | LW | RP | RY | LY | RO | LP |
| 2 | Neutral | LP | RW | RO | LO | RY | RP | LY | LW |
|  | Aggressive | LP | LY | RW | RO | RP | LO | RY | LW |
| 3 | Neutral | RP | LY | RW | LW | LO | RO | LP | RY |
|  | Aggressive | LW | LY | RP | LP | LO | RO | RY | RW |
| 4 | Neutral | RP | RY | RO | RW | LW | LO | LY | LP |
|  | Aggressive | LW | LP | LY | RP | RY | RW | RO | LO |
| 5 | Neutral | RW | RY | LW | RP | LP | LO | LY | RO |
|  | Aggressive | LW | RP | LY | LP | RW | RY | LO | RO |
| 6 | Neutral | RW | RY | LW | LO | LY | RO | RP | LP |
|  | Aggressive | LY | LW | RO | LP | RY | RP | RW | LO |

Note. 8 = best decision, 1 = worst decision. LO = left orange, RO = right orange, LW = left white, RW = right white, LP = left pink, RP = right pink, LY = left yellow, RY = right yellow.

## B. Decision-Making Scoring Criteria for the Field-Based Pre-Post-Test

| Score | Neutral Context | Aggressive Context |
| --- | --- | --- |
| 3 | Participant chose the most conservative option, prioritising the most open player | Participant chose a decision which prioritises moving the ball into the middle of the playing area whilst maintaining ball movement towards the scoring zone |
| 2 | Participant chose an option that was open, irrespective of field placement | Participant chose the next most attacking option, regardless of field position |
| 0 | Any other option | Any other option |
